# Superfamily II helicases: the potential therapeutic target for cardiovascular diseases

**DOI:** 10.3389/fcvm.2023.1309491

**Published:** 2023-12-13

**Authors:** Tianxiang Fang, Xizhi Wang, Ning Huangfu

**Affiliations:** ^1^ Health Science Center, Ningbo University, Ningbo, China; ^2^ Department of Cardiology, The First Affiliated Hospital of Ningbo University, Ningbo, China; ^3^ Department of Cardiology, Key Laboratory of Precision Medicine for Atherosclerotic Diseases of Zhejiang Province, Ningbo, China; ^4^ Clinical Medicine Research Centre for Cardiovascular Disease of Ningbo, Ningbo, China; ^5^ Department of Cardiology, Lihuili Hospital Affiliated to Ningbo University, Ningbo, China

**Keywords:** DEAD-box, RIG-I-like, Brg1, cardiovascular diseases, therapeutic target

## Abstract

Cardiovascular diseases (CVDs) still maintain high morbidity and mortality globally. Helicases, a unique class of enzymes, are extensively implicated in the processes of nucleic acid (NA) metabolism across various organisms. They play a pivotal role in gene expression, inflammatory response, lipid metabolism, and so forth. However, abnormal helicase expression has been associated with immune response, cancer, and intellectual disability in humans. Superfamily II (SFII) is one of the largest and most diverse of the helicase superfamilies. Increasing evidence has implicated SFⅡ helicases in the pathogenesis of multiple CVDs. In this review, we comprehensively review the regulation mechanism of SFⅡ helicases in CVDs including atherosclerosis, myocardial infarction, cardiomyopathies, and heart failure, which will contribute to the investigation of ideal therapeutic targets for CVDs.

## 1. Introduction

Cardiovascular diseases (CVDs) represent a predominant contributor to both morbidity and mortality on a global scale (1, 2). As per the World Health Organization (WHO), in 2019, approximately 17.9 million individuals succumbed to CVD worldwide, constituting 32% of all global fatalities during that year (3, 4). Furthermore, this grim statistic continues to exhibit an upward trajectory annually, with projections indicating an anticipated increase to 23.6 million CVD-related deaths by the year 2030 (2). Particularly in low- and middle-income countries, the absence of organized management for CVDs and their substantial healthcare costs lead to elevated mortality rates when compared to high-income countries (5). Despite notable progress in understanding the pathogenesis of these diseases, there persists an urgent need for more comprehensive research aimed at identifying novel therapeutic targets to mitigate the morbidity and mortality associated with CVDs (6).

A growing body of evidence underscores the pivotal role of SFⅡ helicases in governing the physiological metabolism of organisms (7–9). For instance, studies have revealed the significance of Chromodomain Helicase DNA-binding 4 (CHD4) in facilitating antibody secretion by B lymphocytes in animal models (10). In contrast, the deficiency of SFⅡ helicases is associated with cancer, intellectual disability, premature aging, and immunodeficiency (11). Importantly, it has been early proposed that helicases play an essential role in cardiac development and myocardial infarction (MI) (12, 13). The whole-exome sequencing methods found that the RecQ-like helicase 5 gene was a disease-causing gene of MI (14). Nevertheless, the contributions of SFⅡ helicases to CVDs have yet to be comprehensively delineated. This review, therefore, primarily elucidates the signaling pathways and the involvement of SFⅡ helicases in the context of CVDs. We aim to furnish novel perspectives that can potentially advance the fields of CVD diagnosis, prevention, and therapeutic interventions.

## 2. Helicase

Helicases were initially identified to unwind deoxyribonucleic acid (DNA) or ribonucleic acid (RNA) duplex substrates in a nucleoside triphosphate (NTP) dependent manner (15, 16). Consequently, helicases play essential roles in DNA replication and repair, transcription and translation, RNA synthesis, ribosome synthesis, and so forth (15–17). However, it has become more apparent that helicases are a subgroup of translocases (i.e., move directionally along NA strands by coupling NTP hydrolysis) (17). Nonetheless, not all helicases have translocase activity, such as DEAD-box, and not all helicases have helicase activity, and an example is the Retinoic acid-inducible gene (RIG)-I-like (11).

**Figure 1:**
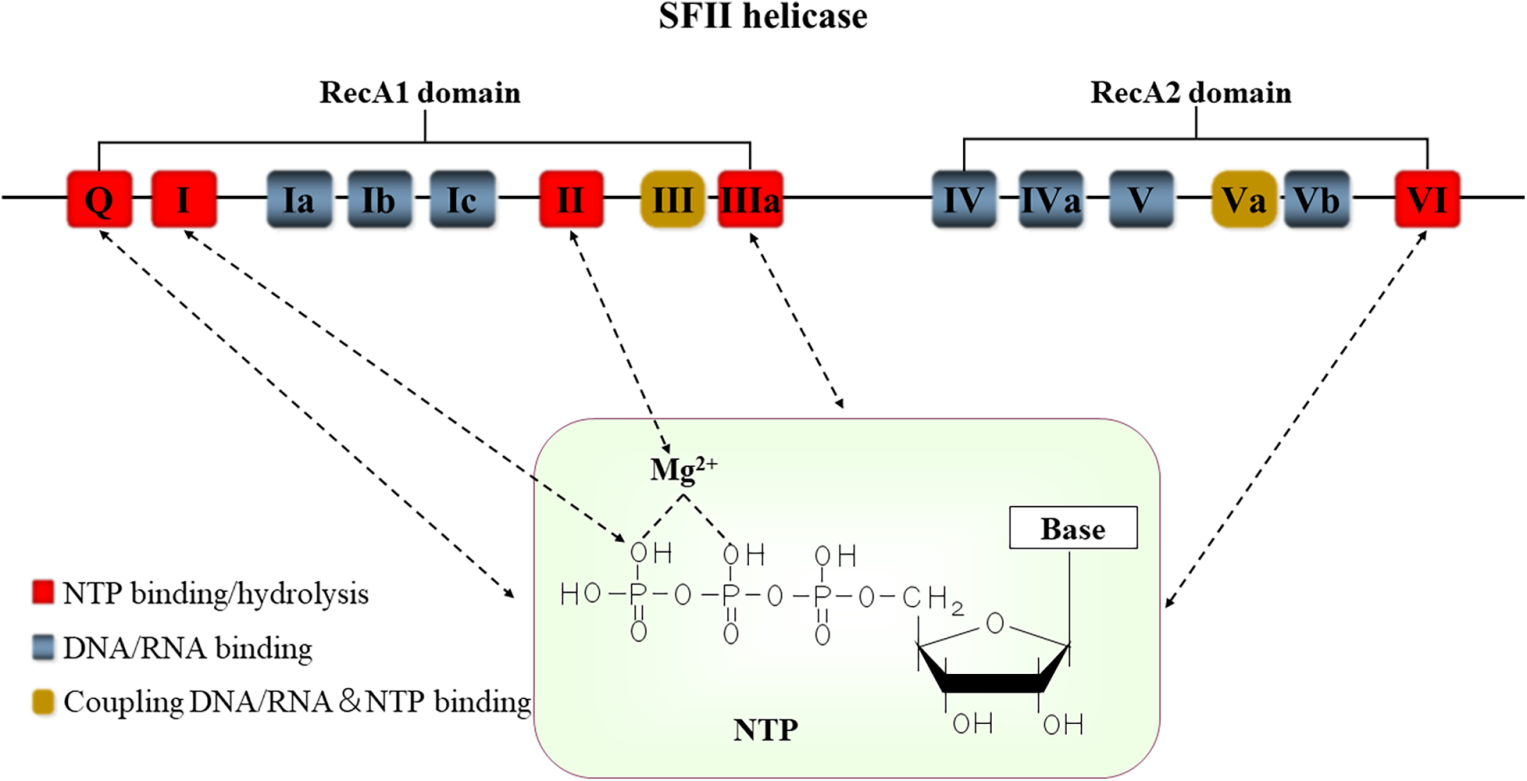
The conserved motifs structure of SFⅡ helicases. Positions of the conserved sequence motifs Q-VI are indicated in this figure. These motifs function as the NTPase activity (red), DNA or RNA sites (blue), and NTP and NA coordinating sites (yellow), respectively. The motif I forms a hydrogen bond with the oxygen atom (O) in the phosphoric acid of the NTP. The motif II contains residues that interact with MgATP/MgADP. Base includes adenine (A), guanine (G), cytosine (C), thymine (T), and uracil (U). NTP: nucleoside triphosphate.

Sequence analysis finds at least fourteen characteristic sequence motifs present by helicase (18). However, not all motifs are present in each helicase (19). Based on these conserved sequence motifs, helicases can be classified into six superfamilies (SFI-SFVI) (17). SFI and SFII have similarly conserved motifs and function as monomers or dimers for unwinding (11, 20). Each monomer is formed from the tandem repeat of two RecA-like folds composed of different motifs (Figure 1) (17, 19). Motifs I and II are also called the phosphate-binding loop or “P-loop” (Walker “A”) and Mg^2+^-binding aspartic acid (Walker “B”) motifs, respectively. As a consequence, they function as NTP binding and hydrolysis and are exclusively shared among all helicases (11, 19, 21). Furthermore, motifs Q, Ia, and VI are also involved in NTP binding and hydrolysis in some helicases. The motif Q is the specific adenosine 5'-triphosphate (ATP)-sensing motif (18, 19). Motifs Ⅰa,Ⅰb, Ⅰc, IV, IVa, Ⅴ, and Ⅴb interacted with NA. Motifs I and Va primarily participate in coordinating NTP and NA binding sites. Once they mutate, it commonly disrupts the coupling of NTP hydrolysis to the binding and unwinding of double-stranded NA (dsNA) (19). In contrast, SFI—SFVI show little similarity to SFI and SFⅡ helicases, function as hexamers or double hexamers, and contain one RecA-like subdomain per monomer (17). SFII is one of the largest and most diverse of the helicase superfamilies, involving almost every process in cells that involves NA metabolism. According to sequence homology, SFⅡ helicases are further divided into ten subfamilies (Table 1): DEAD-box, DEAH/RHA, RIG-I-like, RecQ-like, RecG-like, SWItch/sucrose non-fermentable (Swi/Snf) family, Rad3/XPD, Ski2-like, Type I Restriction Enzyme, NS3/NPH-II (11).

**Table 1:** Classification of SFⅡ helicases.

SFⅡ helicases	Members	Activities	Main function	Refs
DEAD-box	DDX3, DDX5, DDX17, eIF4A3, UAP56	Helicase activity	Participates in all aspects of RNA metabolism.	(11, 23)
DEAH/RHA	Human DHX5, DHX9, DHX30, DHX36, DHX37	Helicase activity Translocase activity	Participates in RNA metabolism.	(11, 23, 24, 28)
RIG-I-like	RIG-I, MDA5, LGP2, Dicer	Translocase activity	Participates in innate immune system and the mature miRNA processing.	(11, 18, 30)
RECQ-like	Human BLM, WRN, RecQ1, RecQ4, RecQ5	Helicase activity Translocase activity	Maintains genomic integrity, such as DNA recombination, telomere maintenance, and DNA damage signaling.	(11)
RECG-like	RecG, PriA	Helicase activity Translocase activity	Participates in prokaryotic DNA metabolism.	(11, 19)
Swi/Snf	SWI/SNF, ISWI, CHD, INO80	Helicase activity Translocase activity	Participates in chromatin structure remodeling.	(11, 37)
Rad3/XPD	Human XPD, FancJ, Rtel1, ChlR1	Helicase activity Translocase activity	Participates in the DNA repair pathways.	(11, 41)
Ski2-like	SKIV2l, MTR4	Helicase activity Translocase activity	Orchestrates 3’-to-5’ RNA decay by the exosome.	(11, 18)
Type I Restriction Enzyme	EcoR124I	Translocase activity	Maintains the host genome integrity and destroys foreign DNA.	(11)
NS3/NPH-II	Hepatitis C virus NS3, Vaccinia virus NPH-II	Helicase activity Translocase activity	Participates in viral replication.	(11)

### 2.1. DEAD-box

DEAD-box, the largest family of SFⅡ helicases, is characterized by a specific amino acid sequence of motif II, Asp-Glu-Ala-Asp (DEAD). All DEAD-box proteins contain a structurally highly conserved core with substrate binding, ATPase activity, and RNA unwinding activity (22). They participate in all aspects of RNA metabolism by disrupting local secondary and tertiary structures of RNA and RNA-protein interactions (11). Over 35 DEAD-box proteins have been identified in humans, such as DDX1, DDX3, DDX5, DDX17, and eukaryotic initiation factor 4A3 (eIF4A3, also known as DDX48) (23). These helicases have a direct link to genome stability. For instance, DDX1 ablation resulted in radiosensitivity and an increase in DNA breaks. DDX3x is an X-linked gene and expression knockdown leads to an accumulation of DNA damage. DDX39B can promote DNA repair gene expression, and deletion causes sensitivity to a series of DNA damaging treatments, such as cisplatin, mitomycin C, and *γ* irradiation. DEAD-box proteins also maintain genome integrity by processing structures such as R-loops (23). Exploring the mechanism of DEAD-box helicases may provide an evolutionary lens on genomic stability.

### 2.2. DEAH/RHA

DEAH/RHA is named for motif II's conserved specific amino acid, Asp-Glu-Ala-His (DEAH), and human RNA helicase A (RHA) (11, 24). They are collectively referred to as DExH-box ATPases, along with RIG-I-like, NS3/NPH-II, and Ski2-like families. DExH represents the amino acid of motif II: Asp-Glu-x-His, where x is any amino acid (25, 26). DEAH/RHA proteins can also separate hybridized RNAs and unwind secondary RNA structures in an NTP-dependent manner. Furthermore, they disrupt RNA-protein interactions with translocate activity, such as Prp43p in yeast (11, 27). DExH-Box helicase 9 (DHX9) triggered innate immune response through the export and translation of retroviral mRNA and recognized microbial NA fragments in the cytoplasm of plasmacytoid dendritic cells (28). Besides the innate immune response, DEAH/RHA helicases are involved in various crucial cellular processes such as nuclear RNA import and export, translational regulation, splicing, and ribosome biogenesis. DHX36 was initially found to facilitate mRNA deadenylation and decay. DHX29 can bind specifically to the 40S ribosomal subunit; it may be associated with translation initiation (28). Extensive research demonstrates that the dysregulation of DEAH/RHA helicases plays a pivotal role in human diseases (24).

### 2.3. RIG-I-like

On the contrary, the RIG-I-like family does not possess the ability to unwind dsRNA. Rather, it exhibits dsRNA translocase activity (11). The RIG-I-like family contains four members: RIG-I, melanoma differentiation associated gene 5 (MDA5), laboratory of genetics and physiology 2 (LGP2), and Dicer (11, 29). Cellularly RIG-I-like receptors (RLRs) are composed of RIG-I, MDA5, and LGP2. In the host's innate immune defense, RLRs trigger the activation of monocytes and macrophages by sensing various damage-associated molecular patterns (DAMPs) (30). Dicer contains an RNase III endonuclease structural domain. Dicer cleaves the precursor microRNAs (pre-miRNAs) (except pre-miR451) into 21–25 nucleotide-long miRNA duplexes in the cytoplasm. Subsequently, one strand of the miRNA duplex generates the RNA-induced silencing complex (RISC), whereas the other strand is typically eliminated and degraded (18, 31, 32). RISC regulates gene expression through degrading mRNA and inhibiting protein translation (31). Emerging research finds that Dicer's DExH domain ensures high-fidelity miRNA biogenesis, and the absence of this domain is lethal for mice (18, 33).

### 2.4. RecQ-like

RecQ-like helicases are DNA helicases with helicase and translocate activities. This family is highly conserved in sequence, structure, and function. They maintain genomic integrity, such as DNA recombination, telomere maintenance, and DNA damage signaling (11). Cells lacking RecQ increased defects in meiosis and chromosome missegregation. There are five members in humans, including Bloom syndrome protein (BLM), Werner syndrome protein (WRN), RecQ1, RecQ4, and RecQ5. Mutations in BLM and WRN are associated with Bloom's syndrome and Werner's syndrome, respectively (11). The RecQL5 gene mutation is the potentially predisposing gene for MI (14). Mutations of RecQ-like helicases are associated with cancer and premature aging, in addition to causing related genetic disorders (11).

### 2.5. RecG-like

RecG-like helicases are involved in prokaryotic DNA metabolism (11). The RecG protein of *Escherichia coli* promotes the rescue of damaged forks by catalyzing Holliday junction recombination with translocation on dsDNA (34). In addition, RecG inhibits DNA replication by unwinding D and R loops (11). Currently, no counterparts resembling RecG-like helicases have been identified within the eukaryotic cells (11, 19, 35). However, several candidates have been proposed, including the human nuclear helicase SMARCAL1. SMARCAL1 is a nuclear DNA damage response protein and travels with the replication fork. Lacking SMARCAL1 is prone to accumulate DNA double-strand breaks (DSBs) in cells (35).

### 2.6. Swi/Snf complexes

Swi/Snf family proteins are ATP-dependent chromatin remodeling complexes and play critical roles in stem cell maintenance, development, and cancer (36). They vary the accessibility of the DNA through remodeling chromatin structure (such as nucleosome structure). They participate in transcription-factor binding, DNA replication and repair (11). Swi/Snf family can be further subdivided into four subfamilies: SWI/SNF, imitation SWI (ISWI), CHD, and INOsitol-requiring mutant 80 (INO80) (36, 37). SWI/SNF is composed of the ATPase subunit Brahma-related gene 1 (Brg1) or Brahma homolog (Brm) and 9–12 Brg1- or Brm-associated factors (Baf) (38). Brg1 and Brm are also SMARCA2 and SMARCA4, respectively, are essential elements of SWI/SNF (39). SWI/SNF protein regulates gene transcription through disrupting nucleosomes; in contrast, ISWI facilitates nucleosome assembly (11). INO80 is the only subfamily with helicase activity involved in DNA repair and transcription. Moreover, CHD regulates chromatin remodeling and transcription activation (11). Multiple studies have highlighted that aberrant chromatin modification is associated with the initiation and progression of tumor behavior (36).

### 2.7. Rad3/XPD

The Rad3 family members include Rad3 (XPD), FancJ, Rtel1, and ChlR1 (11). Utilizing energies from ATP hydrolysis to translocate on single-stranded DNA (ssDNA) and unwind dsDNA, they participate in DNA repair pathways such as nucleotide excision repair (NER), recombinational repair, and homologous recombination (HR) (11). Compared to other DNA damage repair signaling, NER is highly conserved among eukaryotes. Cell NER is impaired, resulting in photosensitivity and high skin cancer risk (40). Additionally, mutations in XPD can cause Xeroderma pigmentosum (XP) and aging diseases (11, 41).

### 2.8. Ski2-like

Ski2-like are RNA helicases and are able to translocate on ssRNA and unwind dsRNA (11, 18). Superkiller Viralicidic Activity 2-Like (SKIV2L) and MTR4 are two Ski-like members in humans with similar functions. They orchestrate 3’-to-5' RNA decay by the exosome. Eukaryotes have nuclear and cytosolic exosomes. SKIV2L acts with both the nuclear and cytosolic exosomes, but MTR4 only with the nuclear exosome. There is an inextricable link between SKIV2L mutations and autoimmunity disease (18).

### 2.9. Type I restriction enzyme

Type I restriction enzymes (TIREs) are present in bacteria and translocate dsDNA powered by ATP hydrolysis. They are large pentameric proteins (R2M2S) consisting of specificity (S), methylase (M), and restriction (R) subunits. TIREs have dual functions that serve to maintain the integrity of the host genome while simultaneously functioning to destroy foreign DNA (11).

### 2.10. NS3/NPH-II

NS3/NPH-II family, encoded by various positive-strand viruses, named from NS3 of hepatitis C virus (HCV) and NPH-II of vaccinia virus. These proteins are essential for viral replication (11). NS3/NPH-II family has dual activities with dsRNA unwind and ssRNA translocating functions. Margaret E et al. found that distinct binding preferences of NPH-II for ssRNA and dsRNA depend on different stages of the ATP hydrolysis cycle. In brief, ADP facilitates the binding between NPH-II and dsRNA. However, there is still poorly understood the mechanism of the unwinding initiation phase for NS3/NPH-II because this mechanic limits the overall unwinding reaction rate (11, 42).

## 3. Role of SFⅡ helicases in AS

### 3.1. Lipid metabolism

Foam cells, the hallmark of early atherosclerosis (AS), are closely linked to the dysregulation of lipid metabolism within macrophages (43). Under normal physiological conditions, there is a dynamic balance between lipid uptake, efflux, and degradation in macrophages. Macrophage scavenger receptor 1 (MSR1) is the major scavenger receptor for binding and uptake of oxidized low-density lipoprotein (ox-LDL). It is highly expressed on the surface of macrophages, and knockdown significantly inhibits foam cell formation (44). In macrophages, ox-LDL stimulated DDX5 expression in a time-dependent manner. Knockdown DDX5 significantly reduced macrophage lipid uptake. Further study found that DDX5 promoted MSR1 protein expression by suppressing the n6-methyladenosyl modification of MSR1 mRNA with the transcription factor mettl3 (45). DDX5 shared a remarkable homology with DDX17 (46). However, DDX17 promoted macrophage cholesterol efflux, not lipid uptake. ATP-binding cassette transporter A1 (ABCA1) is a key transporter protein for macrophage cholesterol efflux. DDX17 protein could interact with and guide the expression of ABCA1. Mechanistic studies revealed that the long noncoding RNA (lncRNA) MeXis established interaction with and directed the promoter binding of the transcriptional coactivator DDX17. It is critical for liver X receptor (LXR)-dependent ABCA1 expression. It is worth noting that DDX17 is also required for maximal ABCA1 expression (47). It is clear that the proteins cannot fully compensate for each other, although there appears to be some functional redundancy between DDX5 and DDX17.

Furthermore, miRNAs also play a vital role in the lipid metabolism of macrophages. For instance, overexpression of miR-155 promoted necrotic core formation by inhibiting macrophages' efferocytosis function (48). In an AS model, knockout of Dicer inhibited the mitochondria fatty acid oxidation (FAO) during macrophage activation, accelerating macrophage lipid load. Dicer promoted macrophage lipid metabolism by inhibiting the corepressors of nuclear receptors (ligand-dependent nuclear receptor corepressor (Lcor) and nuclear receptor corepressor 2 (Ncor2)) *in vitro* through promoting miRNA (such as mi-R10a, Let-7b, and miR-195a) maturation (48).

### 3.2. Inflammatory response

#### 3.2.1. RIG-I-like family

The inflammatory response is involved in all stages of AS and is a pivotal contributor to AS (49). Endothelial cells (ECs), immune cells of the innate and acquired immune system, all regulate the vascular inflammatory response. EC dysfunction and maladaptation are the initiating events of AS (50). Injured ECs trigger pro-inflammatory cytokine secretion and adhesion molecule expression in early AS. Subsequently, immune cells (such as monocytes) are recruited into the subendothelial space. They secrete more inflammatory factors and express more adhesion molecules. These processes activate an inflammatory signaling cascade of AS, ultimately resulting in vascular inflammatory response (50–52). They belonged to RLRs, helicases RIG-I, MDA5, and LGP2, which have been traditionally recognized as constituents of the antiviral immune response. They also contribute to chronic inflammatory diseases like rheumatoid arthritis and lupus nephritis (53). However, in the host, extended and inappropriate immune responses mediate the development of cardiac injury (30). Interferon-γ (IFN-γ) significantly upregulated RIG-I expression in macrophages (54). Moreover, IFN-γ significantly stimulated macrophage interferon regulatory factor 1 (IRF1) expression, which was associated with M1 macrophage polarization (55). Another research investigation documented the IRF1/RIG-I axis mediated 25-hydroxycholesterol-induced interleukin-8 (IL-8) secretion. It implied that IFN-γ/IRF1/RIG-I axis might be essential in differentiating and activating macrophages in AS. The activation of RIG-I could stimulate several downstream transcription factors, including nuclear factor Kappa-beta (NF-κB), activator protein-1 (AP-1), and nuclear factor interleukin-6 (NF-IL-6) through mitochondrial antiviral signaling protein/transforming growth factor-beta-activated kinase 1 (MAVS/TAK-1) signaling pathways (56). Utilizing bioinformatics analysis, Ruoyu Dong et al. highlighted that MDA5 might be one of the hub genes potentially associated with the immunity of AS (57). Moreover, in human coronary artery endothelial cells (HCAEC), overexpression of RIG-I and MDA5 were linked with intercellular adhesion molecule-1 (ICAM1) and vascular cell adhesion molecule 1 (VCAM1) activation, pro-inflammatory cytokines [IL-6, IL-8, IFN-γ–induced protein 10 (IP-10)] secretion, and cellular reactive oxygen species (ROS) production (53, 58). Intriguingly, MDA5 could further induce EC apoptosis, whereas RIG-I stimulation had no such effects (53). Taken together, these studies demonstrated that RIG-I and MDA5 activation led to AS by enhancing macrophage and EC inflammation pathways and dysfunction.

EC pyroptosis and apoptosis play distinct roles in inflammatory response (52). Dicer is also involved in inflammatory response by regulating miRNA biogenesis in AS. Anti-inflammatory IL-32α inhibited EC inflammation by suppressing the Rprd2-Dgcr8/DDX5-Dicer1 axis. This axis downregulated expression of the tissue inhibitor of metalloproteinase 3 (Timp3) and reversion-inducing cysteine-rich protein with Kazal motifs (Reck) by promoting miR-205 biogenesis. Timp3 and Reck are endogenous vascular protective genes that function as AS protective genes in ECs (50). Moreover, Dicer promoted EC maladaptation and chemokine expression by regulating miR-103. Monocytes were recruited and entered into the impaired intima with endothelial chemokines, facilitating foam cell formation (32). However, it was in contrast to the role of Dicer in macrophages. These discrepancies might, in part, be due to Dicer targeting different miRNAs in the different cells. For example, miR-10a, miR-146a, and miR-181b induced anti-inflammatory phenotype, whereas miR-19a had a pro-inflammatory effect in ECs (32). However, the mechanism of how Dicer selectively targets specific pre-miRNAs remains unclear; it requires further research.

#### 3.2.2. DEAD-box and DEAH/RHA family

DHX9 has been reported to trigger inflammation and complications such as AS in the sera of systemic lupus erythematosus patients (59). Recently, a study reported for the first time that DHX9 was highly expressed in the peripheral blood mononuclear cells (PBMCs) of patients with coronary artery disease. Knockdown of DHX9 significantly ameliorated the development of AS in ApoE -/- mice fed with a Western diet. Further investigation revealed that activation of the nucleus DHX9-p65-RNA Polymerase II complex triggered inflammatory factor transcriptional expression (59). Thus, it is imperative to consider targeting DHX9 as a therapeutic strategy for the treatment of AS. Moreover, overexpression of eIF4A3 enhanced Gasdermin D (GSDMD) stability in ECs, then promoted GSDMD expression. The eIF4A3 is the exon junction complex (EJC) core protein with the RNA splicing function. The mRNA by EJC spliced showed higher stability and translation (60). The activation of GSDMD triggered EC pyroptosis under caspase 1/4/5 activation (52).

#### 3.2.3. Swi/Snf family

More literature supports that the Swi/Snf family plays a vital role in the inflammation of AS. Fei Fang et al. found that Brg1 and Brm significantly promoted the expression of adhesion molecules through NF-κB/p65 in ECs (61). Similarly, Yuanyuan Zhang et al.'s study showed that knockout of Brg1 downregulated the expression of EC inflammatory factors and chemokines by limiting c-Fos expression and, subsequently, nucleic translocation (51). Brg1 interacted with the sequence-specific transcription factor Egr-1 and upregulated the expression of EC Spondin 2 (SPON2). SPON2 functioned as pattern recognition receptors (PRRs) to regulate immunity response (62). Moreover, overexpression of Brg1 was associated with vascular homeostasis. Brg1 interacted with the transcription factor EST1 to induce PR65A expression. PR56A inhibited endothelial nitric oxide synthase (eNOS) activity and NO bioavailability. Limited NO bioavailability induced EC injury and accelerated the development of AS (63). Remarkably, the previous study by Fei Fang et al. suggested that the expression of Baf47 in human aortic artery endothelial cells (HAECs) remained essentially unchanged after treatment with ox-LDL. Recently, another investigation illustrated an augmentation in the interaction between Baf47 and cAMP response element binding protein 1 (CREB1) upon pro-inflammatory stimulation, which complied with Fei Fang et al.'s result. Baf47-CREB1 complex cooperatively enhanced the transcriptional activation of Neogenin 1 (Neo1). Neo1 shared a remarkable homology with PRRs and promoted adhesion factor expression through the NF-κB signaling pathway (64). These pieces of evidence highlight that SWI/SNF-associated subunit activation contributes to EC inflammatory and vascular homeostasis. Further research on the detailed mechanisms of SWI/SNF in AS-associated inflammation is warranted.

### 3.3. Vascular calcification

Vascular calcification is also a significant cause of AS. Although RLRs have traditionally been regarded as part of the immune response, the mutations of RIG-I and MDA5 mediated the development of vascular calcification in Singleton-Merten syndrome (an autosomal-dominant multi-system disorder) (65, 66). Emerging studies have shown that RIG-I could promote osteogenic signals in aortic vascular smooth muscle cells (VSMCs). Knockout RIG-I reduced GTPase-activating protein-binding protein (G3BP1) Arg methylation, resulting in downstream osteogenic mineralization response (67) (Table 2).

**Table 2:** The association between SFⅡ helicases and atherosclerosis.

SFⅡ helicases	Targeted genes	Functions	Refs
Lipid metabolism			
DDX5	MSR1	Promotes macrophage uptake ox-LDL.	(45)
DDX17	ABCA1	Promotes macrophage cholesterol efflux.	(47)
Dicer	mi-R10a, Let-7b, miR-195a	Promotes macrophage mitochondria fatty acid oxidation.	(48)
Inflammatory response			
RIG-I	NF-κB, AP-1, NF-IL-6	Promotes macrophage IL-8 secretion.	(56)
RIG-I/MDA5	unknown	Promotes EC expressing ICAM1, VCAM1, IL-6, IL-8, IP-10 and ROS.	(53, 58)
MDA5	unknown	Promotes EC apoptosis.	(53)
Dicer/DDX5	Timp3/ Reck	Promotes EC inflammation.	(50)
Dicer	miR-103	Promotes EC maladaptation and chemokine expression.	(32)
DHX9	NF-κB	Promotes macrophage inflammatory factor secretion.	(59)
eIF4A3	GSDMD	Promotes EC pyroptosis.	(52)
Brg1/Brm	NF-κB	Promotes EC adhesion molecule expression.	(61)
Brg1	c-Fos	Promotes EC inflammatory factor and chemokine expression.	(51)
	SPON2	Promotes EC inflammation.	(62)
	eNOS	Promotes EC injury.	(63)
Baf47	Neo1	Promotes EC adhesion molecule expression.	(64)
Vascular calcification			
RIG-I	G3BP1	Promotes VSMC osteogenic mineralization responses.	(67)

## 4. Role of SFⅡ helicases in MI and ischemia/reperfusion injury (IRI)

### 4.1. MI

Acute myocardial infarction (AMI) primarily results from reduced or closed blood flow in a life-threatening portion of coronary vessels (68). Especially in coronary arteries with combined AS unstable plaque rupture with subsequent thrombosis is a common clinical cause of AMI. The helicase eIF4A3 overexpression increased the risk of advanced plaque rupture (60). In vitro, eIF4A3-siRNA reduced mRNA stability and protein expression of forkhead box O1 (FOXO1) and beclin1, resulting in abnormal autophagy (69).

AMI induces cardiomyocyte ischemia and hypoxia, then ultimately leads to irreversible damage, necrosis, and apoptosis of cardiomyocytes. Downregulation of Brg1 expression induced hypoxia-induced cell oxidative stress and injuries in H9C2 cells. Inverse regulations were found in cells with Brg1 expression upregulation (68). Brg1 inhibited cardiomyocyte injury and apoptosis through activating downstream phosphatidyl inositol 3-kinase/AKT/the mammalian target of rapamycin (PI3K/AKT/mTOR) and Nuclear erythroid 2-related factor 2/ Heme oxygenase 1 (Nrf2/HO-1) signaling pathways (68, 70). Brg1 also functions as a significant factor in promoting neovascularization and cardiomyogenesis in MI. It occurs through the reactivation of the fetal gene program in epicardium-derived cells (EPDCs). A vitro study showed that Brg1 interacted with Thymosin b4 (Tb4), promoting optimal transcription of Wilms' tumour 1 (Wt1), which is the master regulator of embryonic EPDCs (71). Overall, Brg1 protected cardiac injury from AMI. More importantly, it restored cardiac function by promoting neovascularization and cardiomyogenesis. However, the reactivation of fetal genes may induce cardiac pathologic hypertrophy (72). Moreover, Brg1 has been reported to be associated with hypertrophic cardiomyopathy (HCM) and heart failure (HF) pathological remodeling mechanisms (73, 74). Brg1 reactivated in the adult myocardium, in this setting, may promote pathology and adverse remodeling rather than the regenerative response.

### 4.2. IRI

**Figure 2:**
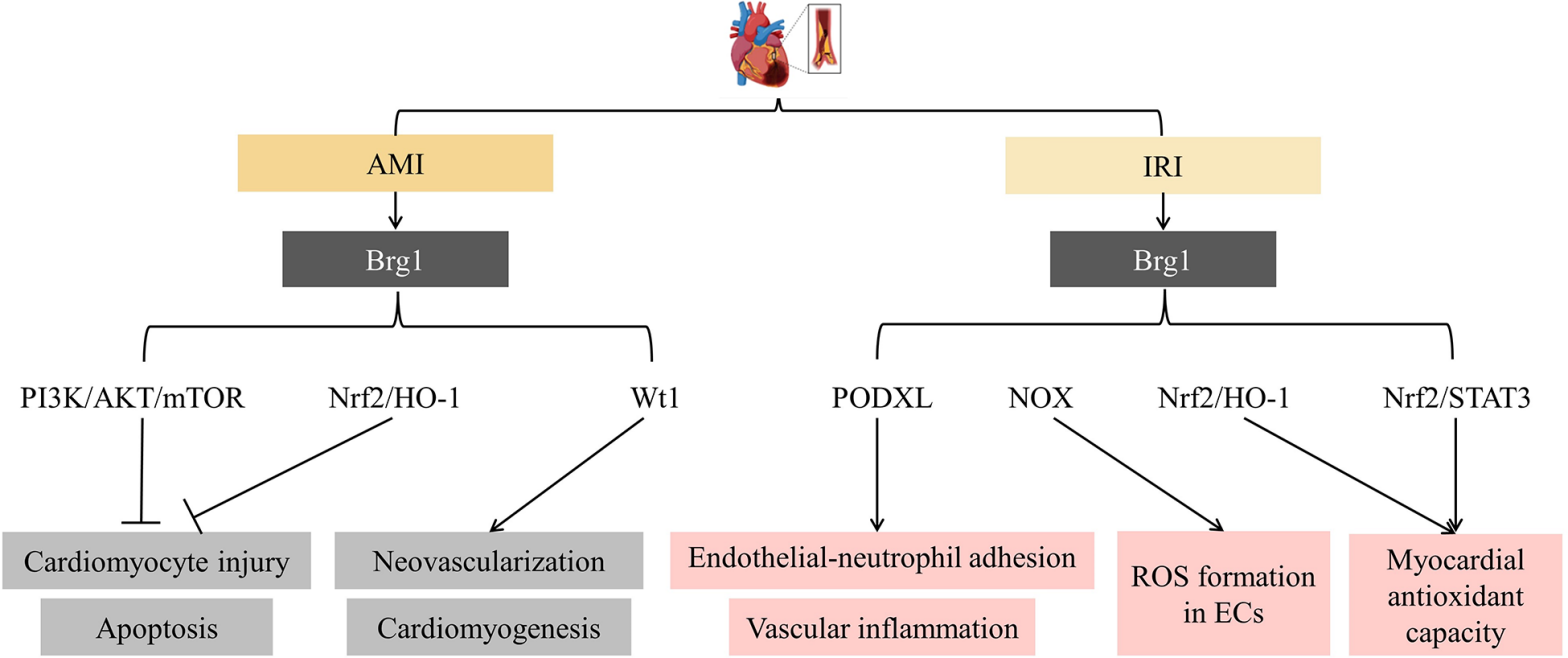
SFⅡ helicases in myocardial infarction (MI) and ischemia/reperfusion injury (IRI). It showed that Brg1 inhibited cardiomyocyte injury and apoptosis and promoted neovascularization and cardiomyogenesis in MI. However, it facilitated endothelial cell (EC) inflammatory response and the development of IRI. PI3K: phosphatidyl inositol 3-kinase; mTOR: the mammalian target of rapamycin; Nrf2: Nuclear erythroid 2-related factor 2; HO-1: Heme oxygenase 1; Wt1: Wilms’ tumour 1; PODXL: podocalyxin; NOX: the NADPH oxidases; STAT3: Signal transducer and activator of transcription-3; ROS: reactive oxygen species.

Early thrombolytic therapy and percutaneous coronary intervention (PCI) have been effective in reducing mortality in MI. However, heart reperfusion may result in further cardiac damage, usually named IRI (75). It is associated with the elevated recruitment and adhesion of circulating leukocytes, specifically neutrophils, to the vessel wall after reperfusion. The infiltration leukocyte, in turn, contributes to myocardial injury by triggering ROS production and the inflammatory cascade response (76). Xinjian Zhang et al. and Zilong Li et al. identified Brg1 as a molecule implicated in the pathogenesis of IRI in ECs (76, 77). The former found that Brg1 promoted endothelial-neutrophil adhesion and vascular inflammation by stimulating the transcriptional of podocalyxin (PODXL, a neutrophil ligand). Endothelial-specific Brg1 knockout mice attenuated cardiac fibrosis and better-recovered heart function after long-term (6 weeks) IRI stimulation than the control (76). Based on it, another scholar proposed that the metastasis-associated lung adenocarcinoma transcript 1 (MALAT1)/miR-144/Brg1 signaling pathway might also mediate the inflammation response of myocardial IRI (78). Zilong Li et al.'s study showed that Brg1 promoted the NADPH oxidases (NOX) transcription, thereby mediating EC ROS formation (77). However, as mentioned previously, Brg1 had a protective role in preventing cardiomyocyte injury from AMI. Cardiomyocyte Brg1 downregulation meditated cellular susceptibility to oxidative stress in diabetic patients who are experiencing IRI. Further investigation into the underlying mechanisms revealed that inhibiting the Brg1/Nrf2/HO-1 and Brg1/Nrf2/Signal transducer and activator of transcription-3 (STAT3) signaling pathways reduced the myocardial antioxidant capacity (75, 79) (Figure 2).

The discoveries above support the essential roles of Brg1 in regulating myocardial oxidative stress and injuries, highlighting that it is a promising therapeutic strategy for treating AMI/IRI (80). In addition, given that Brg1 could promote EC inflammation, how to activate myocardial Brg1 selectively is a challenge in MI/IRI.

## 5. Role of SFⅡ helicases in the cardiomyopathies

Cardiomyopathies are a group of heterogeneous myocardial diseases. Various reasons cause mechanical and/or electrical dysfunction of the heart. Based on predominant organ involvement, cardiomyopathies are classified into two main groups: primary and secondary cardiomyopathies. Primary cardiomyopathies are mainly restricted to the heart muscle and are relatively few. They can be further subclassified as genetic, mixed (genetic and nongenetic), and acquired primary cardiomyopathies. In contrast, secondary cardiomyopathies manifest as pathological myocardial involvement. They are primarily associated with generalized systemic (multiorgan) disorders (e.g., infections, metabolic diseases, and endocrine diseases) (81).

### 5.1. Primary cardiomyopathies

#### 5.1.1. Genetic

HCM is the most prevalent form of genetic primary cardiomyopathies. It is caused by mutations encoding contractile proteins of the cardiac sarcomere. The β-myosin heavy chain (β-MHC, also known as MYH7) is one of the predominant disease-causing genes (81). In adults, cardiomyocytes majorly express α-myosin heavy chain (α-MHC, also known as MYH6), not β-MHC. However, on abnormal stress, cardiac shifted from α-MHC to β-MHC expression and induced pathological hypertrophy on abnormal stress (72). Immunohistochemical staining from 796 human cardiac specimens found that Brg1 expression significantly increased in patients with HCM compared to other causes of ventricular hypertrophy and dilated cardiomyopathy (DCM). In vitro, inhibition of Brg1 significantly decreased cardiomyocyte β-MHC expression and increased α- MHC expression (73), consistent with findings reported by Hang et al. (72). However, the evidence of Brg1 as the causative gene for HCM is remained limited. Numerous questions remain, such as Brg1 is vital for physiologic remodeling during fetal cardiac development but closes in adults. It reactivated in the adult is associated with diseases.

#### 5.1.2. Mixed (genetic and nongenetic)

Mixed primary cardiomyopathies are comprised of DCM and primary restrictive nonhypertrophied cardiomyopathy. DCM is the leading cause of heart transplantation. It is characterized by ventricular dilatation with impaired progressive systolic diastole. Pathologically, it is associated with viral infection, inflammatory response, and aberrant genetic expression (81). Cardiac Dicer and RHAU deficiency might participate in the progression of DCM (82). Dicer expression level significantly decreased in human patients with DCM. Dicer mutant mice exhibited severe left ventricular dilatation and a dramatic decrease in systole. It was attributed to miRNA maturation abnormality (83). Furthermore, RHAU mediated dual regulation of mRNA translation and stability. RHAU facilitated mRNA translation by binding to the 5'-UTR of mRNA and promoted mRNA degradation with the 3'-UTR of mRNA. The anatomical and histological analysis of the hearts found that RHAU conditional knockout postnatal mice exhibited progressive DCM. In vitro, silenced RHAU destabilized the mRNA of hexamethylene bis-acetamide inducible 1/yes1associated transcriptional regulator/NK2 homeobox 5 (Hexim1/Yap1/Nkx2-5). These mRNAs play crucial roles in heart regeneration and function (82).

Numerous prior studies have investigated the mRNA and miRNA as integral players in the cardiovascular system's physiological and pathophysiological processes (84, 85). It is necessary to investigate further the relationship between SFⅡ helicases (such as Dicer and RHAU) and miRNA and mRNA in mixed primary cardiomyopathies, including DCM.

#### 5.1.3. Acquired

Acquired cardiomyopathies include myocarditis, stress (“Tako-Tsubo”) cardiomyopathy, and peripartum (postpartum) cardiomyopathy. Myocarditis is an acute or chronic inflammatory cardiomyopathy caused by various reasons (mainly microbial infections such as viruses) (81). Several immune cells, such as monocytes and macrophages, are in the heart tissue. They have double functions: promoting heart inflammation and fibrosis, supporting heart repair after cardiac injury, and reducing inflammation and infection. The activation of monocytes and macrophages is initiated by various DAMPs that target specific PRRs, including RLRs (30). RLRs initiated antiviral response by inducing phosphorylation of NF-κB and IRF3 (30). In a mouse model of Coxsackievirus B3 (CVB3)-induced myocarditis, cardiac-specific overexpression of MDA5 attenuated cardiac viral replication, cardiomyocyte injury, and apoptosis. It emphasized that MDA5 inhibited viral proliferation through IRF3-dependent antiviral type I IFN pathways (86). It is consistent with Yumei Han et al., who found that MDA5 induced antiviral factor IFN-β expression partially through MAVS/TBK1/IRF3 signaling pathways (87). However, another study suggested that the activation of MDA5 and RIG-I induced human cardiac fibroblasts (CF) to produce high amounts of IL-6 and IL-8 (30). Persistent activation of RLRs may mediate the progression of severe myocarditis. Further investigations will be required to understand how to regulate RLRs exert their physiological functions. In addition, Dicer, another RIG-I-like family member, could also inhibit cardiomyocyte apoptosis by promoting the expression of miRNA-222 in the viral myocarditis model (88).

### 5.2. Secondary cardiomyopathies

Secondary cardiomyopathies are comparatively complex in number and causes, including infection, metabolize dysfunction, endocrine disorders, and toxicity (81). HO-1 functions as anti-apoptotic and anti-inflammatory properties. In diabetes patients, heart HO-1 expression downregulation is a vital pathogenic mechanism of diabetic cardiomyopathy. Numerous research confirmed that hyperglycemia stimulation inhibited Brg1/Nrf2/HO-1 signaling pathways and impaired heart antioxidant capacity (75, 79, 89). In vitro, upregulation of Brg1 expression attenuated hyperglycemia-induced cardiomyocyte oxidative stress and apoptosis (89). These findings highlight that Brg1 induces cardiomyocyte oxidative stress through mediating Nrf2/HO-1 signaling pathways.

**Figure 3:**
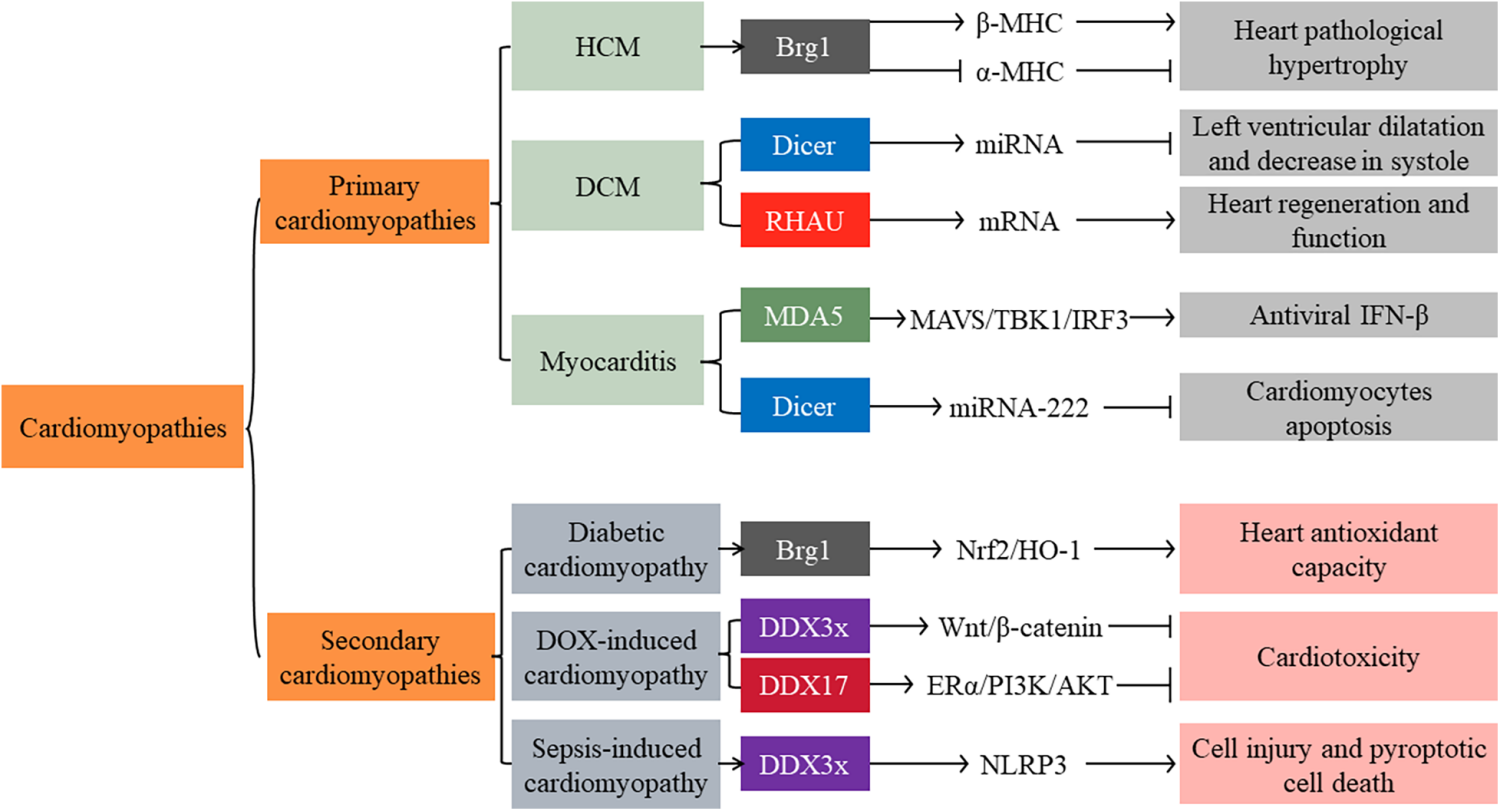
Main signaling pathways of SFⅡ helicases involved in primary and secondary cardiomyopathies. HCM: Hypertrophic cardiomyopathy; DCM: dilated cardiomyopathy; DOX: doxorubicin; β-MHC: β-myosin heavy chain; α-MHC: α-myosin heavy chain; MAVS: mitochondrial antiviral signaling protein; TBK1: TANK-Binding Kinase 1; IRF: interferon regulatory factor; ERα: estrogen receptor α; NLRP3: NOD-like receptor protein 3; IFN: interferon.

In addition, DEAD-box family members DDX3x and DDX17 have been proven to play a protective role in doxorubicin (DOX)-induced cardiotoxicity. In vitro, overexpression DDX3x and DDX17 ameliorated Dox-induced cardiomyocyte injury and apoptosis. Mechanistic studies have shown that they prevented cardiotoxicity by activating Wnt/β-catenin and estrogen receptor α (ERα)/PI3K/Akt signaling, respectively (90, 91). However, DDX3x might play deleterious roles in sepsis-induced cardiomyopathy. In vitro simulation sepsis, cardiomyocyte DDX3x expression significantly increased. DDX3x promoted NOD-like receptor protein 3 (NLRP3) inflammasome assembly and induced cell injury and pyroptotic cell death. However, only *in vitro* data were provided in this study (92). More detailed mechanisms of SFⅡ helicases remain largely unknown. Further studies are required to confirm whether these proteins may be a potential therapeutic target for secondary cardiomyopathies (Figure 3).

## 6. Role of SFⅡ helicases in VSMC-associated vascular diseases

VSMCs are the major cellular components of aortic media. Aberrant proliferation and migration, phenotypic transition (from contractile to synthetic phenotypic), and apoptosis are all involved in the progression of vascular diseases (93, 94).

### 6.1. Vascular development and remodeling

Dicer-dependent miRNAs were essential for normal VSMC proliferation and differentiation. Deleting VSMC Dicer resulted in dilated and thin-walled blood vessels due to cellular proliferation reduction. VSMC-specific knockout Dicer mice exhibited impaired vascular contractility (31, 95). Furthermore, helicase DDX5 was the most abundantly expressed DEAD-box protein in human arteries, mainly located in VSMCs (96). Overexpression of DDX5 inhibited VSMC aberrant proliferation and migration. Consequently, it markedly attenuated pathological vascular remodeling and prevented vascular stenosis, especially in injured arteries. In vitro, DDX5 directly interacted with GATA-binding protein 6 (GATA-6) and maintained its expression. This complex, in turn, repressed aberrant proliferation and migration of VSMCs by elevating the transcription of p27^Kip1^ (96). In addition, the DDX5-serum response factor (SRF) axis was impaired and also participated in VSMC dedifferentiation and vascular remodeling (97). On the other hand, Abha Sahni et al. identified UAP56 as a potential target for treating vascular proliferative disease. Knockdown of UAP56 in VSMCs inhibited transcriptional activation of E2F, a cell cycle regulator, consequently suppressing Ang II-induced cell DNA synthesis and proliferation (98, 99). These findings highlighted DDX5's and UAP56's potential therapeutic benefit in injury-related arterial disease.

### 6.2. Hypertension

VSMC's phenotypic transition from contractile to synthetic phenotype was strongly related to the pathogenesis of hypertension. In the peripheral blood from essential hypertension (EH) patients, CHD1-Like (CHD1L) expression significantly increased compared to the control group. In vitro, downregulating CHD1L expression inhibited Ang II-induced cell phenotypic transition, proliferation, migration, and inflammation response (100). Similarly, the overexpression of CHD9, another member of the CHD family, served to enhance VSMC phenotypic transition, proliferation, migration, and oxidative stress (101). Further studies are required to better define CHD proteins' function and fundamental mechanisms in hypertension.

### 6.3. Aortic aneurysm

Abdominal aortic aneurysm (AAA) is an irreversible cardiovascular disease. The prevalence is about 1.3% in women over 65 years of age, whereas in men, this figure reaches 5%, according to previous statistics. What is terrifying is that the mortality of AAA ruptured is between 50% and 80% (102). Multiterm studies demonstrated that VSMC apoptosis, extracellular matrix (ECM) degradation, and inflammatory response contributed to the progression of AAA (102, 103). However, the molecular mechanisms of AAA development and progression still need to be completed.

Recent attention has focused on SFⅡ helicases that might participate in developing AAA (51, 104, 105). Baf60a expression significantly increased in patients with AAA. VSMC-specific knockout Baf60a inhibited inflammatory response and ECM degradation in AAA mice. In vitro, Baf60a deficiency inhibited pro-inflammation leukocyte factors secretion and cathepsin S (CTSS) expression. The CTSS played a crucial role in the degradation of ECM and contributed to vascular inflammation. Baf60a knockout blocked Brg1 recruitment to the promoter region of NF-κB target genes (104). Furthermore, Brg1 expression increased in the aortic media of thoracic aortic aneurysm (TAA) patients. Overexpression of Brg1 promoted VSMC apoptosis, indicating it might also be involved in the development of TAA (106, 107). Tianming Le et al. found recently that the zeste homolog 2 (EZH2)-mediated RIG-I signaling pathway promoted VSMC apoptosis and AAA development. Last but not least, DDX3x protein was also upregulated in AAA. Overexpression of DDX3x activated VSMC AKT pathways. It mediated cell ROS activation, pro-inflammatory cytokines release, and phenotypic transition (103).

Collectively, SFⅡ helicases are associated with a variety of inflammatory diseases. The density of inflammatory cells in the aortic epicardium was positively correlated with the diameter of the aneurysm. It is significant to further investigate the regulation mechanisms of SFⅡ helicases in the AAA.

### 6.4. Aortic dissection

**Figure 4:**
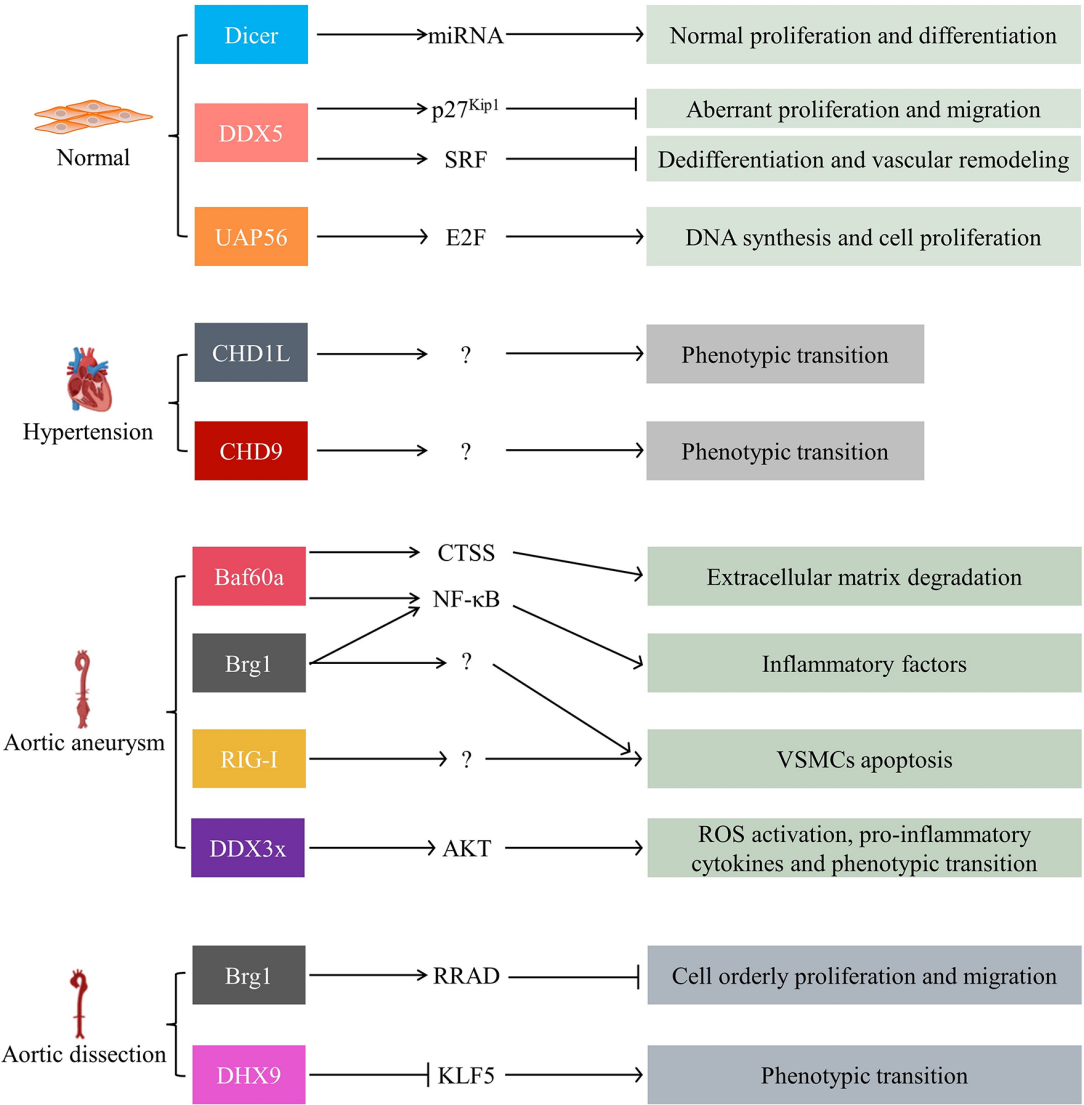
SFⅡ helicases in VSMC-associated vascular diseases, including vascular development and remodeling, hypertension, aortic aneurysm, and aortic dissection. SRF: serum response factor; CTSS: cathepsin S; NF-κB: nuclear factor Kappa-beta; RRAD: Ras-related associated with diabetes; KLF5: the Krüppel-like factor; VSMCs: vascular smooth muscle cells.

Aortic dissection (AD) is a vascular disease with a high mortality. Impaired VSMCs' structure and function are intensely associated with AD development. In the VSMCs with AD patients, Brg1 expression was positively correlated to cell Matrix metalloproteinases 2 (MMP2) and MMP9 expression, cell apoptosis, and phenotypic transition (from contractile to synthetic phenotype) (93). Similar to the discoveries presented by Wei-Lin Liao et al., Brg1 directly upregulated the expression of Ras-related associated with diabetes (RRAD). RRAD functioned as an inhibitor of orderly proliferation and migration in VSMCs (94). In contrast, DHX9 exhibited a protective role, as observed in a study. SMC-specific knockout DHX9 further exacerbated the transition of VSMCs from a contractile phenotype to a synthetic phenotype. The mechanistic study showed that DHX9 might induce alternative splicing of the Krüppel-like factor (KLF) 5 mRNA by bridging YB-1. This process reduced KLF5 stability and nuclear localization and attenuated the VSMCs phenotype transition (108) (Figure 4).

## 7. Role of SFⅡ helicases in cardiac remodeling and HF

HF is a clinical syndrome consequence of the progression of CVDs. It features ventricular dilatation and myocardial contractility decrease. HF due to pathologic cardiac hypertrophy is the leading cause of mortality worldwide (109). Increasing research showed that SFⅡ helicases are closely associated with HF. In this respect, it had been reported that eIF4A3 played a prominent role in myocyte and heart function. The eIF4A3 is crucial for mRNA stability and translation. There was significant relocalization of eIF4A3 from the nucleus to the cytoplasm in hypoxia-induced metabolic stress. Small changes in the expression and distribution of this nucleocytoplasmic shuttling protein will have profound implications for mRNA processing and potential adaptation to stress. Knockdown eIF4A3 expression significantly disrupted cardiomyocyte contractility and structure in response to metabolic stress (60). However, this result was contradictory to Qi-rong Xu et al.'s findings, which presented the nucleus circCmss1/eIF4A3/ transferrin receptor 1 (TfR1) axis-induced cardiomyocyte ferroptosis and promoted ventricular remodeling upon the Nuclear SET Domain 2 (NSD2) stimulation (110). This differential effect might be caused by eIF4A3 subcellular distribution differences.

**Figure 5:**
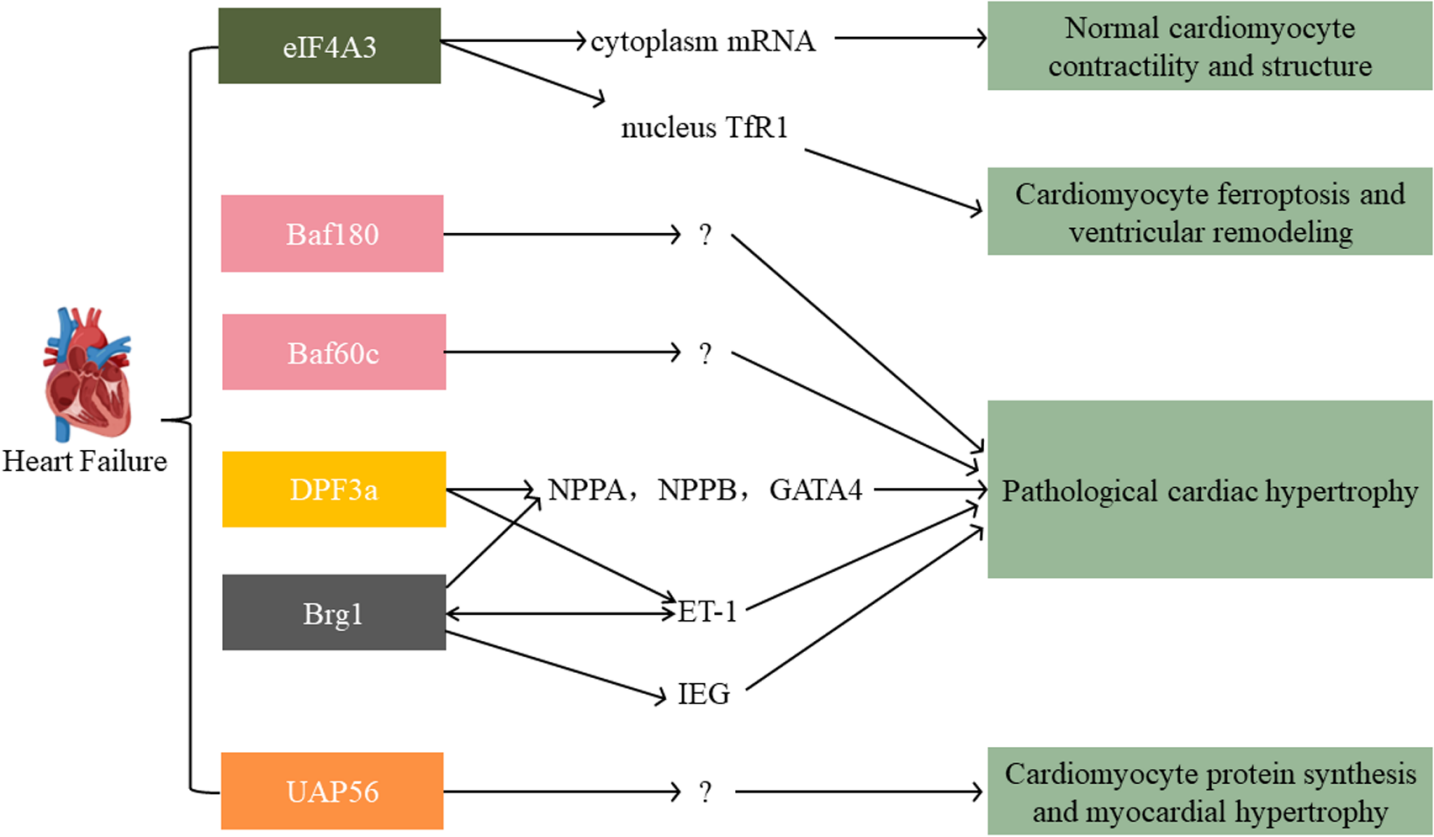
Main signaling pathways of SFⅡ helicases involved in heart failure. The eIF4A3 has an inverse role in the nucleus vs. the cytoplasm. SWI/SNF family (Baf180, Baf60c, DPF3, Brg1) and UAP56 all exert an acceleration function in pathological cardiac hypertrophy. TfR1: transferrin receptor 1; NPPA/NPPB: natriuretic peptides; GATA4: GATA binding protein 4; ET-1: endothelin-1; IEG: immediate early gene.

Furthermore, Brg1, Baf180, and Baf60c were significantly expressed in hypertrophic hearts. Chromatin immunoprecipitation (CHIP) analysis showed that these three proteins' recruitment to the atrial natriuretic peptide (ANP) and brain natriuretic peptide (BNP) promoters were elevated (74). ANP and BNP are highly expressed in fetal ventricles and crucial for developing cardiac and then turning off at birth, but they reactivate upon hypertrophy stimulation (74). Based on a transverse aortic constriction (TAC) model, ANP and BNP expression increased confirmed the development of cardiac hypertrophy. Brg1 expression was significantly increased at 8 weeks after TAC. It implied that Brg1 might play a predominant role in increasing the expression of these two hypertrophy markers. In vitro, the depletion of Brg1 abrogated isoproterenol-induced increase in cell size and expression of ANP and BNP mRNA. Brg1 knockdown partially abrogated the microphthalmia-associated transcription factor (MITF) association with GATA binding protein 4 (GATA4) promoter. GATA4 is a critical transcriptional regulator of BNP and ANP (38). However, the knockdown of Brg1 in unstimulated cardiomyocytes did not affect cell size in this study. Indicating Brg1 might regulate distinct genes in unstimulated and hypertrophic cardiomyocytes. In addition, Brg1 by DPF3a (Baf45c) recruitment will abolish HEY inhibition. Under physiological conditions, HEY, a transcriptional repressor, binds the promoters of genomic targets like natriuretic peptides (NPPA, NPPB) and GATA4. However, upon hypertrophic stimuli, Brg1 and DPF3a physically interact and release HEY from the genomic targets, inducing cardiac hypertrophy. Surprisingly, deleting DPF3 could buffer endothelin-1 (ET-1) induced hypertrophy in this study (111, 112). ET-1 is an endothelium-derived pluripotent factor that is fundamental in developing pathological hypertrophic remodeling (112). In line with this, Emma L. Robinson et al. found that the mitogen and stress-activated protein kinase/Brg1/immediate early gene (MSK/Brg1/IEG) axis mediated ET-1 initiated cardiac hypertrophy (113). In addition, Brg1 and Brm could be the upstream factors of ET-1, promoting its expression in a time-dependent manner (112). These studies implied that SWI/SNF subfamily helicases might be critical in cardiac hypertrophy and HF, especially ET-1-associated. Moreover, Abha Sahni et al. found that UAP56 functioned as a regulator of cardiomyocyte protein synthesis. It could induce myocardial hypertrophy in response to neurohumoral stimulation (114) (Figure 5).

Currently, the drug investigation has largely been successful in delaying HF progression. However, the outcome of the disease was greatly limited by the fact that HF patients are often combined with other systemic diseases, such as chronic kidney disease, diabetes, and depression. Moreover, the drugs themselves often lead to adverse effects (109). Studies of the epigenetic mechanisms of HF will promise to provide inspiration for new therapeutic drugs.

## 8. Conclusions

Therefore, the identification of diverse classifications of helicases associated with the development and progression of disease is of great importance in providing new therapeutic targets for clinical treatment.

SFⅡ helicases yield a pivotal regulatory influence across a broad spectrum of CVDs, including AS, MI, and HF. However, the role of the same helicase is distinct in different cells and exhibits a heterogeneous role even in the same tissue (48, 50, 60, 110). Furthermore, a multitude of issues and hurdles persist in the clinical utilization of helicases as therapeutic targets for addressing CVDs: (1) whether the specific roles of helicases in humans are consistent with *in vitro* and animal experiments are yet unknown; (2) whether there is positive and/or negative feedback regulation or synergy between each helicase, like DDX5 and Dicer1 in AS; (3) how to target and regulate helicase expression in specific tissues and organs. Comprehensive investigations into the roles of helicases remain imperative, as they hold the potential to offer novel perspectives on the diagnosis, treatment, and even the prognostication of not only CVDs but also various other ailments.
